# Chlorido[5-meth­oxy-1*H*-benzimidazole-2(3*H*)-thione-κ*S*]bis­(tri­phenyl­phos­phane-κ*P*)copper(I) methanol disolvate

**DOI:** 10.1107/S1600536814001251

**Published:** 2014-01-22

**Authors:** Qing-Xuan Meng

**Affiliations:** a Daxing High School Attached to CNU, Beijing 102600, People’s Republic of China

## Abstract

In the title complex, [CuCl(C_8_H_8_N_2_OS)(C_18_H_15_P)_2_]·2CH_3_OH, the Cu^I^ ion is coordinated by one chloride anion, one S atom from the 5-meth­oxy-1*H*-benzimidazole-2(3*H*)-thione ligand and two P atoms from two tri­phenyl­phosphine ligands in a distorted tetra­hedral geometry. One of the N-bound H atoms is involved in an intra­molecular N—H⋯Cl hydrogen bond, while another one inter­acts with the solvent methanol mol­ecule *via* an N—H⋯O hydrogen bond. Inter­molecular O—H⋯Cl and O—H⋯O hydrogen bonds link two further complex mol­ecules and four solvent mol­ecules into a centrosymmetric structural unit. The short distance of 3.624 (4) Å between the centroids of the five- and the six-membered rings of two benzimidazole fragments indicates the presence of π–π inter­actions.

## Related literature   

For the structures and properties of Cu^I^ complexes with triphenlyphosphine ligands, see: Gennari *et al.* (2006 ▶); Kitagawa *et al.* (1995 ▶); Raper (1994 ▶). For complexes with a 5-meth­oxy-1*H*-benzimidazole-2(3*H*)-thione ligand, see: Schneider *et al.* (2008 ▶). For related structures, see: Lobana & Castineiras (2002 ▶).
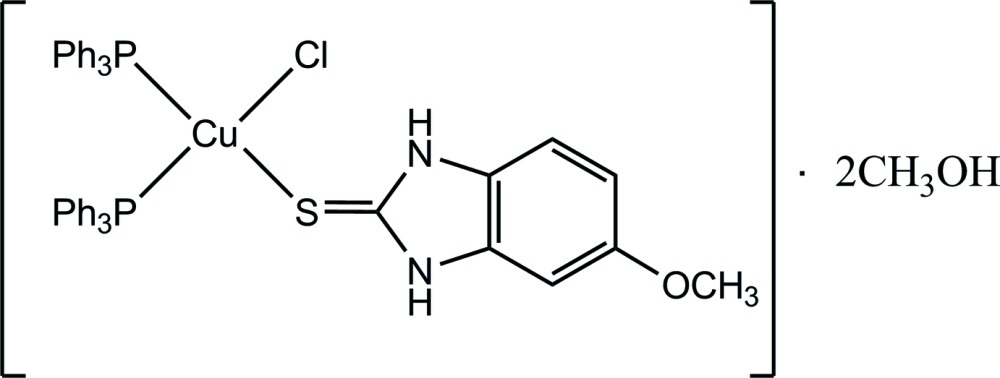


## Experimental   

### 

#### Crystal data  

[CuCl(C_8_H_8_N_2_OS)(C_18_H_15_P)_2_]·2CH_4_O
*M* *_r_* = 867.84
Monoclinic, 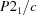

*a* = 12.8354 (9) Å
*b* = 18.4979 (17) Å
*c* = 18.7933 (18) Å
β = 92.839 (12)°
*V* = 4456.6 (7) Å^3^
*Z* = 4
Mo *K*α radiation
μ = 0.71 mm^−1^
*T* = 298 K
0.34 × 0.27 × 0.15 mm

#### Data collection  

Bruker SMART APEX CCD area-detector diffractometer
Absorption correction: multi-scan (*SADABS*; Bruker, 2007 ▶) *T*_min_ = 0.794, *T*_max_ = 0.901
22332 measured reflections
7835 independent reflections
3113 reflections with *I* > 2σ(*I*)
*R*_int_ = 0.143

#### Refinement  

*R*[*F* ^2^ > 2σ(*F* ^2^)] = 0.086
*wR*(*F* ^2^) = 0.217
*S* = 1.07
7835 reflections
510 parameters
H-atom parameters constrained
Δρ_max_ = 1.53 e Å^−3^
Δρ_min_ = −0.57 e Å^−3^

### 

Data collection: *SMART* (Bruker, 2007 ▶); cell refinement: *SAINT-Plus* (Bruker, 2007 ▶); data reduction: *SAINT-Plus*; program(s) used to solve structure: *SHELXS97* (Sheldrick, 2008 ▶); program(s) used to refine structure: *SHELXL97* (Sheldrick, 2008 ▶); molecular graphics: *SHELXTL* (Sheldrick, 2008 ▶); software used to prepare material for publication: *SHELXTL*.

## Supplementary Material

Crystal structure: contains datablock(s) global, I. DOI: 10.1107/S1600536814001251/cv5442sup1.cif

Structure factors: contains datablock(s) I. DOI: 10.1107/S1600536814001251/cv5442Isup2.hkl

Supporting information file. DOI: 10.1107/S1600536814001251/cv5442Isup3.cdx

CCDC reference:

Additional supporting information:  crystallographic information; 3D view; checkCIF report

## Figures and Tables

**Table 1:** Hydrogen-bond geometry (Å, °)

*D*—H⋯*A*	*D*—H	H⋯*A*	*D*⋯*A*	*D*—H⋯*A*
N1—H1⋯Cl1	0.86	2.30	3.136 (6)	165
N2—H2⋯O2	0.86	2.08	2.893 (9)	157
O2—H2*A*⋯O3^i^	0.82	2.00	2.728 (10)	148
O3—H3⋯Cl1^ii^	0.82	2.35	3.170 (8)	176

Symmetry codes: (i) 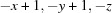; (ii) 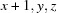.

**Fig. 1.**
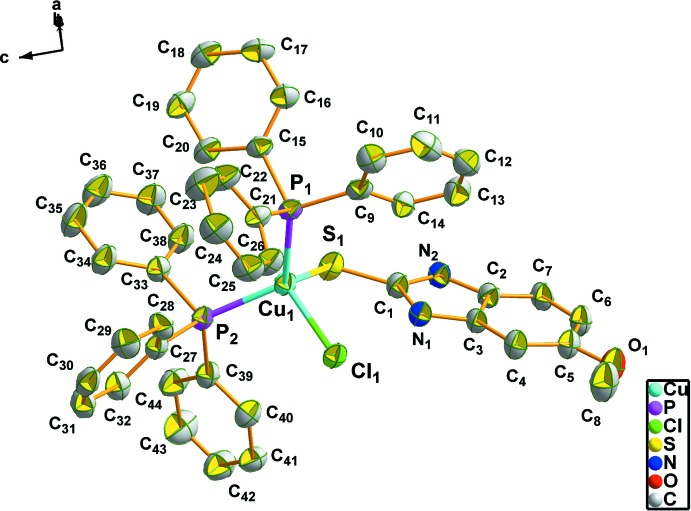
The molecular structure of the title compound showing the atomic numbering and 30% probability displacement ellipsoids. Solvent molecules and H atoms have been omitted for clarity.

## supplementary crystallographic information

### 1. Comment

Cu(I) complexes containing triphenlyphosphine and mercaptan ligands have received much attention in the past, mainly because of their interesting coordination chemistry and potential applications in photography, biochemistry and enzymatic reactions (Gennari *et al.*, 2006; Kitagawa *et al.*, 1995; Raper *et al.*, 1994). However, only one structure was reported for metal-MOBMT complex (MOBMT = 5-methoxy-1*H*- benzimidazole-2(3*H*)-thione) (Schneider *et al.*, 2008). Herewith we present the crystal structure of new Cu(I) complex with triphenlyphosphine and MOBMT ligands.

In the title complex, MOMBT act as neutral, monodentate ligand with the S atom as a coordination atom. Other sites of the coordination tetrahedron are occupied by two P atoms from two triphenlyphosphine ligands and one halide anion. The Cu—S and Cu—P bond distances are similar to those reported in other copper(I) complexes (Lobana *et al.*, 2002). The environment around copper(I) is distorted tetrahedral, angles around the Cu atom ranging from 102.1 (1)–122.0 (1)°. A dimer is formed by hydrogen bonds N—H···O, O—H···Cl, O—H···O between the unit [CuX(thione)(PPh_3_)_2_] and the solvent methanol molecules. An intramolecular N—H···Cl hydrogen bond is also observed (Table 1). Furthermore, the centroid to centroid distance between the parallel five- and six-membered rings of two benzimidazole fragments is 3.624 (4) Å, which suggests an existence of π···π interactions between them.

### 2. Experimental

A mixture of CuCl (0.2 mmol) and 5-methoxy-1*H*-benzimidazole- 2(3*H*)-thione(0.2 mmol) in MeOH and CH_2_Cl_2_ (10 mL, *v*/*v* = 1:1) was stirred for 2 h and triphenylphosphine (0.2 mmol) was added to the mixture which was stirred for another 4 h. The insoluble residues were removed by filtration, and filtrate was evaporated slowly at room temperature for two weeks to yield colorless crystalline products.

### 3. Refinement

H atoms were positioned geometrically [C—H = 0.93 – 0.96 Å, N—H = 0.86 Å, O—H = 0.82 Å] and refined as riding, with *U*_iso_(H) = 1.2 – 1.5 *U*_eq_ of the parent atom.

### Crystal data

**Table:**

[CuCl(C_8_H_8_N_2_OS)(C_18_H_15_P)_2_]·2CH_4_O	*F*(000) = 1808
*M**_r_* = 867.84	*D*_x_ = 1.293 Mg m^−^^3^
Monoclinic, *P*2_1_/*c*	Mo *K*α radiation, λ = 0.71073 Å
Hall symbol: -P 2ybc	Cell parameters from 1965 reflections
*a* = 12.8354 (9) Å	θ = 2.6–18.0°
*b* = 18.4979 (17) Å	µ = 0.71 mm^−^^1^
*c* = 18.7933 (18) Å	*T* = 298 K
β = 92.839 (12)°	Block, colorless
*V* = 4456.6 (7) Å^3^	0.34 × 0.27 × 0.15 mm
*Z* = 4	

### Data collection

**Table:**

Bruker SMART APEX CCD area-detector diffractometer	7835 independent reflections
Radiation source: fine-focus sealed tube	3113 reflections with *I* > 2σ(*I*)
Graphite monochromator	*R*_int_ = 0.143
phi and ω scans	θ_max_ = 25.0°, θ_min_ = 2.6°
Absorption correction: multi-scan (*SADABS*; Bruker, 2007)	*h* = −15→15
*T*_min_ = 0.794, *T*_max_ = 0.901	*k* = −15→21
22332 measured reflections	*l* = −22→22

### Refinement

**Table:**

Refinement on *F*^2^	Primary atom site location: structure-invariant direct methods
Least-squares matrix: full	Secondary atom site location: difference Fourier map
*R*[*F*^2^ > 2σ(*F*^2^)] = 0.086	Hydrogen site location: inferred from neighbouring sites
*wR*(*F*^2^) = 0.217	H-atom parameters constrained
*S* = 1.07	*w* = 1/[σ^2^(*F*_o_^2^) + (0.0574*P*)^2^] where *P* = (*F*_o_^2^ + 2*F*_c_^2^)/3
7835 reflections	(Δ/σ)_max_ = 0.001
510 parameters	Δρ_max_ = 1.53 e Å^−^^3^
0 restraints	Δρ_min_ = −0.57 e Å^−^^3^

### Special details

**Table:**

Geometry. All e.s.d.'s (except the e.s.d. in the dihedral angle between two l.s. planes) are estimated using the full covariance matrix. The cell e.s.d.'s are taken into account individually in the estimation of e.s.d.'s in distances, angles and torsion angles; correlations between e.s.d.'s in cell parameters are only used when they are defined by crystal symmetry. An approximate (isotropic) treatment of cell e.s.d.'s is used for estimating e.s.d.'s involving l.s. planes.
Refinement. Refinement of *F*^2^ against ALL reflections. The weighted *R*-factor *wR* and goodness of fit *S* are based on *F*^2^, conventional *R*-factors *R* are based on *F*, with *F* set to zero for negative *F*^2^. The threshold expression of *F*^2^ > σ(*F*^2^) is used only for calculating *R*-factors(gt) *etc*. and is not relevant to the choice of reflections for refinement. *R*-factors based on *F*^2^ are statistically about twice as large as those based on *F*, and *R*- factors based on ALL data will be even larger.

### Fractional atomic coordinates and isotropic or equivalent isotropic displacement parameters (Å^2^)

**Table:**

	*x*	*y*	*z*	*U*_iso_*/*U*_eq_	
Cu1	0.30121 (7)	0.43378 (5)	0.23518 (5)	0.0537 (3)	
P1	0.47643 (15)	0.42103 (10)	0.21637 (10)	0.0509 (5)	
P2	0.24071 (15)	0.44918 (10)	0.34688 (10)	0.0531 (6)	
Cl1	0.22504 (15)	0.32234 (10)	0.18410 (10)	0.0599 (5)	
S1	0.22509 (17)	0.53539 (11)	0.17178 (11)	0.0692 (7)	
N1	0.1747 (4)	0.4387 (3)	0.0660 (3)	0.0563 (16)	
H1	0.1900	0.4013	0.0915	0.068*	
N2	0.1489 (5)	0.5499 (3)	0.0335 (3)	0.0628 (18)	
H2	0.1446	0.5963	0.0348	0.075*	
O1	0.0716 (5)	0.3451 (4)	−0.1723 (3)	0.0926 (19)	
O2	0.1114 (6)	0.6996 (4)	−0.0042 (5)	0.130 (3)	
H2A	0.0639	0.6951	−0.0347	0.195*	
O3	0.9931 (7)	0.2825 (5)	0.1330 (5)	0.158 (4)	
H3	1.0532	0.2907	0.1475	0.236*	
C1	0.1816 (6)	0.5083 (4)	0.0894 (4)	0.059 (2)	
C2	0.1233 (6)	0.5082 (4)	−0.0262 (4)	0.058 (2)	
C3	0.1393 (6)	0.4368 (4)	−0.0056 (4)	0.0524 (19)	
C4	0.1246 (6)	0.3797 (4)	−0.0514 (4)	0.065 (2)	
H4	0.1378	0.3324	−0.0368	0.078*	
C5	0.0892 (6)	0.3957 (5)	−0.1203 (5)	0.066 (2)	
C6	0.0695 (6)	0.4672 (5)	−0.1402 (4)	0.070 (2)	
H6	0.0441	0.4766	−0.1865	0.084*	
C7	0.0856 (6)	0.5234 (5)	−0.0950 (4)	0.065 (2)	
H7	0.0719	0.5707	−0.1096	0.079*	
C8	0.1079 (9)	0.2732 (6)	−0.1583 (5)	0.122 (4)	
H8A	0.1639	0.2744	−0.1225	0.182*	
H8B	0.1324	0.2526	−0.2013	0.182*	
H8C	0.0518	0.2444	−0.1418	0.182*	
C9	0.5097 (7)	0.4059 (4)	0.1244 (4)	0.059 (2)	
C10	0.6037 (7)	0.3743 (5)	0.1044 (5)	0.082 (3)	
H10	0.6544	0.3605	0.1388	0.099*	
C11	0.6198 (9)	0.3640 (5)	0.0318 (7)	0.094 (3)	
H11	0.6812	0.3426	0.0180	0.113*	
C12	0.5476 (11)	0.3847 (6)	−0.0176 (6)	0.095 (3)	
H12	0.5605	0.3775	−0.0653	0.115*	
C13	0.4570 (9)	0.4156 (6)	−0.0013 (6)	0.096 (3)	
H13	0.4079	0.4295	−0.0367	0.116*	
C14	0.4393 (7)	0.4258 (4)	0.0702 (5)	0.072 (2)	
H14	0.3770	0.4472	0.0822	0.087*	
C15	0.5550 (6)	0.4989 (4)	0.2451 (5)	0.059 (2)	
C16	0.6271 (6)	0.5323 (5)	0.2041 (5)	0.073 (2)	
H16	0.6379	0.5151	0.1586	0.088*	
C17	0.6840 (7)	0.5918 (5)	0.2304 (6)	0.085 (3)	
H17	0.7320	0.6139	0.2019	0.102*	
C18	0.6702 (7)	0.6179 (5)	0.2974 (6)	0.084 (3)	
H18	0.7088	0.6572	0.3147	0.101*	
C19	0.5988 (7)	0.5855 (5)	0.3385 (5)	0.082 (3)	
H19	0.5885	0.6028	0.3841	0.098*	
C20	0.5415 (6)	0.5266 (5)	0.3126 (5)	0.073 (2)	
H20	0.4929	0.5053	0.3411	0.088*	
C21	0.5458 (6)	0.3451 (4)	0.2602 (4)	0.058 (2)	
C22	0.6389 (7)	0.3535 (5)	0.2985 (5)	0.088 (3)	
H22	0.6673	0.3994	0.3055	0.106*	
C23	0.6913 (8)	0.2926 (6)	0.3271 (6)	0.109 (4)	
H23	0.7547	0.2983	0.3526	0.131*	
C24	0.6496 (8)	0.2252 (5)	0.3176 (5)	0.089 (3)	
H24	0.6857	0.1849	0.3354	0.106*	
C25	0.5562 (8)	0.2171 (5)	0.2826 (5)	0.084 (3)	
H25	0.5263	0.1714	0.2779	0.101*	
C26	0.5054 (7)	0.2761 (5)	0.2539 (4)	0.072 (2)	
H26	0.4414	0.2694	0.2293	0.086*	
C27	0.2655 (6)	0.3748 (4)	0.4116 (4)	0.057 (2)	
C28	0.3502 (7)	0.3323 (4)	0.4029 (4)	0.070 (2)	
H28	0.3908	0.3396	0.3639	0.084*	
C29	0.3773 (7)	0.2775 (5)	0.4521 (5)	0.084 (3)	
H29	0.4360	0.2489	0.4466	0.100*	
C30	0.3162 (8)	0.2672 (5)	0.5073 (5)	0.079 (3)	
H30	0.3334	0.2310	0.5402	0.095*	
C31	0.2305 (8)	0.3080 (5)	0.5166 (5)	0.081 (3)	
H31	0.1894	0.2996	0.5551	0.098*	
C32	0.2046 (7)	0.3622 (4)	0.4682 (5)	0.072 (2)	
H32	0.1455	0.3902	0.4742	0.087*	
C33	0.2920 (6)	0.5294 (4)	0.3946 (4)	0.061 (2)	
C34	0.3381 (7)	0.5259 (5)	0.4634 (5)	0.081 (3)	
H34	0.3414	0.4823	0.4881	0.097*	
C35	0.3790 (8)	0.5885 (6)	0.4946 (5)	0.103 (3)	
H35	0.4107	0.5863	0.5402	0.123*	
C36	0.3736 (8)	0.6538 (6)	0.4593 (6)	0.101 (3)	
H36	0.3992	0.6955	0.4815	0.121*	
C37	0.3302 (7)	0.6571 (5)	0.3913 (6)	0.089 (3)	
H37	0.3290	0.7006	0.3665	0.106*	
C38	0.2885 (7)	0.5960 (5)	0.3598 (5)	0.080 (3)	
H38	0.2571	0.5991	0.3142	0.095*	
C39	0.0997 (6)	0.4619 (4)	0.3492 (4)	0.063 (2)	
C40	0.0361 (7)	0.4197 (4)	0.3041 (4)	0.074 (2)	
H40	0.0664	0.3882	0.2726	0.089*	
C41	−0.0717 (7)	0.4236 (5)	0.3052 (5)	0.081 (3)	
H41	−0.1129	0.3936	0.2757	0.097*	
C42	−0.1163 (8)	0.4702 (7)	0.3483 (6)	0.103 (3)	
H42	−0.1886	0.4728	0.3486	0.123*	
C43	−0.0571 (9)	0.5136 (6)	0.3914 (6)	0.115 (4)	
H43	−0.0890	0.5469	0.4203	0.138*	
C44	0.0514 (8)	0.5090 (5)	0.3931 (5)	0.091 (3)	
H44	0.0913	0.5381	0.4243	0.109*	
C45	0.1070 (11)	0.7687 (8)	0.0263 (7)	0.184 (6)	
H45A	0.0960	0.7643	0.0763	0.277*	
H45B	0.0505	0.7954	0.0036	0.277*	
H45C	0.1714	0.7936	0.0199	0.277*	
C46	0.9510 (12)	0.2268 (10)	0.1738 (8)	0.231 (10)	
H46A	0.9228	0.2470	0.2158	0.346*	
H46B	1.0049	0.1928	0.1874	0.346*	
H46C	0.8967	0.2026	0.1461	0.346*	

### Atomic displacement parameters (Å^2^)

**Table:**

	*U*^11^	*U*^22^	*U*^33^	*U*^12^	*U*^13^	*U*^23^
Cu1	0.0613 (6)	0.0469 (6)	0.0522 (6)	−0.0002 (5)	−0.0049 (4)	−0.0005 (5)
P1	0.0540 (12)	0.0396 (12)	0.0584 (13)	−0.0011 (10)	−0.0033 (10)	0.0018 (10)
P2	0.0631 (13)	0.0440 (13)	0.0516 (13)	0.0016 (10)	−0.0029 (10)	−0.0023 (10)
Cl1	0.0783 (13)	0.0395 (11)	0.0605 (13)	−0.0042 (10)	−0.0093 (10)	−0.0004 (9)
S1	0.0936 (16)	0.0418 (12)	0.0695 (15)	0.0030 (11)	−0.0228 (12)	0.0021 (11)
N1	0.074 (4)	0.038 (4)	0.056 (4)	0.002 (3)	−0.011 (3)	0.005 (3)
N2	0.074 (4)	0.055 (5)	0.057 (4)	0.001 (4)	−0.009 (3)	0.012 (4)
O1	0.122 (5)	0.075 (5)	0.078 (4)	0.021 (4)	−0.020 (4)	−0.010 (4)
O2	0.162 (8)	0.073 (5)	0.150 (7)	−0.005 (5)	−0.045 (5)	0.023 (5)
O3	0.140 (7)	0.146 (8)	0.178 (8)	−0.042 (6)	−0.081 (6)	0.048 (6)
C1	0.065 (5)	0.053 (6)	0.059 (6)	0.003 (4)	−0.006 (4)	0.005 (5)
C2	0.063 (5)	0.054 (6)	0.055 (6)	0.002 (4)	−0.008 (4)	0.010 (5)
C3	0.068 (5)	0.039 (5)	0.049 (5)	0.003 (4)	−0.006 (4)	0.006 (4)
C4	0.082 (6)	0.051 (5)	0.062 (6)	0.012 (5)	−0.011 (5)	0.004 (5)
C5	0.082 (6)	0.060 (6)	0.055 (6)	0.009 (5)	−0.007 (5)	−0.002 (5)
C6	0.088 (6)	0.068 (6)	0.053 (6)	0.012 (5)	−0.009 (4)	0.005 (5)
C7	0.082 (6)	0.057 (6)	0.057 (6)	0.007 (5)	−0.005 (5)	0.014 (5)
C8	0.190 (11)	0.079 (8)	0.094 (8)	0.007 (8)	−0.014 (8)	−0.016 (6)
C9	0.069 (6)	0.036 (5)	0.073 (6)	−0.009 (4)	−0.001 (5)	0.001 (4)
C10	0.094 (7)	0.066 (6)	0.088 (8)	0.007 (5)	0.007 (6)	0.000 (5)
C11	0.122 (10)	0.060 (7)	0.103 (9)	0.001 (6)	0.045 (8)	−0.009 (6)
C12	0.127 (10)	0.078 (8)	0.084 (9)	−0.028 (8)	0.025 (8)	−0.018 (7)
C13	0.116 (9)	0.096 (8)	0.077 (8)	−0.017 (7)	−0.002 (6)	−0.010 (6)
C14	0.085 (6)	0.071 (6)	0.062 (6)	−0.011 (5)	0.010 (5)	−0.013 (5)
C15	0.055 (5)	0.048 (5)	0.072 (6)	−0.002 (4)	−0.004 (4)	0.003 (4)
C16	0.078 (6)	0.056 (6)	0.086 (7)	−0.006 (5)	0.004 (5)	−0.002 (5)
C17	0.078 (7)	0.064 (7)	0.113 (9)	−0.015 (5)	0.010 (6)	−0.002 (6)
C18	0.080 (7)	0.065 (7)	0.107 (9)	−0.006 (5)	−0.013 (6)	−0.010 (6)
C19	0.085 (7)	0.068 (7)	0.093 (7)	−0.003 (5)	−0.005 (6)	−0.024 (5)
C20	0.072 (6)	0.062 (6)	0.085 (7)	−0.005 (5)	−0.009 (5)	−0.007 (5)
C21	0.056 (5)	0.046 (5)	0.073 (6)	0.001 (4)	−0.001 (4)	−0.006 (4)
C22	0.086 (7)	0.054 (6)	0.121 (8)	−0.003 (5)	−0.020 (6)	0.009 (6)
C23	0.105 (8)	0.080 (8)	0.139 (10)	0.006 (7)	−0.042 (7)	0.023 (7)
C24	0.104 (8)	0.047 (6)	0.113 (8)	0.013 (6)	−0.010 (6)	0.015 (6)
C25	0.098 (8)	0.048 (6)	0.106 (8)	0.001 (6)	0.001 (6)	0.005 (5)
C26	0.083 (6)	0.043 (5)	0.086 (6)	0.005 (5)	−0.016 (5)	0.000 (5)
C27	0.073 (6)	0.042 (5)	0.056 (6)	0.001 (4)	−0.005 (4)	−0.003 (4)
C28	0.085 (6)	0.059 (6)	0.067 (6)	0.002 (5)	0.006 (5)	0.014 (5)
C29	0.091 (7)	0.068 (7)	0.091 (8)	0.011 (5)	−0.008 (6)	0.025 (6)
C30	0.103 (8)	0.062 (6)	0.069 (7)	−0.010 (6)	−0.027 (6)	0.012 (5)
C31	0.114 (8)	0.066 (7)	0.064 (6)	−0.005 (6)	0.004 (6)	0.008 (5)
C32	0.092 (7)	0.058 (6)	0.067 (6)	0.005 (5)	0.006 (5)	−0.003 (5)
C33	0.077 (6)	0.047 (5)	0.060 (6)	0.002 (4)	0.003 (4)	−0.005 (4)
C34	0.109 (7)	0.056 (6)	0.076 (7)	−0.008 (5)	−0.015 (6)	−0.010 (5)
C35	0.139 (9)	0.075 (8)	0.089 (8)	−0.010 (7)	−0.037 (6)	−0.011 (6)
C36	0.126 (9)	0.064 (8)	0.109 (9)	−0.010 (6)	−0.019 (7)	−0.025 (7)
C37	0.128 (8)	0.045 (6)	0.092 (8)	−0.006 (6)	−0.011 (6)	−0.004 (5)
C38	0.106 (7)	0.052 (6)	0.079 (6)	−0.006 (5)	−0.013 (5)	−0.009 (5)
C39	0.075 (6)	0.053 (5)	0.061 (6)	0.004 (5)	−0.001 (5)	−0.005 (4)
C40	0.076 (7)	0.064 (6)	0.080 (6)	0.006 (5)	−0.005 (5)	−0.009 (5)
C41	0.064 (6)	0.086 (7)	0.092 (7)	−0.011 (6)	−0.003 (5)	−0.008 (6)
C42	0.079 (7)	0.113 (9)	0.116 (9)	0.001 (7)	−0.001 (7)	−0.015 (7)
C43	0.093 (9)	0.119 (10)	0.133 (10)	0.018 (8)	0.014 (7)	−0.045 (8)
C44	0.082 (8)	0.091 (8)	0.099 (8)	−0.001 (6)	0.001 (6)	−0.025 (6)
C45	0.219 (16)	0.153 (15)	0.176 (14)	−0.013 (12)	−0.046 (11)	−0.046 (12)
C46	0.225 (17)	0.24 (2)	0.215 (17)	−0.121 (16)	−0.117 (14)	0.093 (15)

### Geometric parameters (Å, º)

**Table:**

Cu1—P2	2.292 (2)	C19—C20	1.389 (11)
Cu1—P1	2.306 (2)	C19—H19	0.9300
Cu1—S1	2.407 (2)	C20—H20	0.9300
Cu1—Cl1	2.456 (2)	C21—C22	1.373 (10)
P1—C9	1.822 (8)	C21—C26	1.382 (10)
P1—C15	1.825 (8)	C22—C23	1.405 (12)
P1—C21	1.835 (8)	C22—H22	0.9300
P2—C39	1.828 (8)	C23—C24	1.365 (12)
P2—C33	1.838 (8)	C23—H23	0.9300
P2—C27	1.853 (8)	C24—C25	1.348 (11)
S1—C1	1.695 (8)	C24—H24	0.9300
N1—C1	1.362 (8)	C25—C26	1.368 (11)
N1—C3	1.398 (8)	C25—H25	0.9300
N1—H1	0.8600	C26—H26	0.9300
N2—C1	1.352 (8)	C27—C28	1.359 (10)
N2—C2	1.387 (9)	C27—C32	1.371 (10)
N2—H2	0.8600	C28—C29	1.404 (10)
O1—C5	1.363 (9)	C28—H28	0.9300
O1—C8	1.429 (10)	C29—C30	1.345 (11)
O2—C45	1.403 (13)	C29—H29	0.9300
O2—H2A	0.8200	C30—C31	1.351 (11)
O3—C46	1.410 (14)	C30—H30	0.9300
O3—H3	0.8200	C31—C32	1.383 (11)
C2—C7	1.387 (10)	C31—H31	0.9300
C2—C3	1.388 (9)	C32—H32	0.9300
C3—C4	1.370 (10)	C33—C38	1.394 (11)
C4—C5	1.382 (10)	C33—C34	1.398 (10)
C4—H4	0.9300	C34—C35	1.388 (12)
C5—C6	1.395 (10)	C34—H34	0.9300
C6—C7	1.352 (10)	C35—C36	1.380 (13)
C6—H6	0.9300	C35—H35	0.9300
C7—H7	0.9300	C36—C37	1.370 (12)
C8—H8A	0.9600	C36—H36	0.9300
C8—H8B	0.9600	C37—C38	1.371 (11)
C8—H8C	0.9600	C37—H37	0.9300
C9—C14	1.378 (10)	C38—H38	0.9300
C9—C10	1.409 (11)	C39—C44	1.370 (11)
C10—C11	1.403 (12)	C39—C40	1.387 (10)
C10—H10	0.9300	C40—C41	1.387 (10)
C11—C12	1.336 (13)	C40—H40	0.9300
C11—H11	0.9300	C41—C42	1.332 (12)
C12—C13	1.344 (13)	C41—H41	0.9300
C12—H12	0.9300	C42—C43	1.348 (13)
C13—C14	1.387 (11)	C42—H42	0.9300
C13—H13	0.9300	C43—C44	1.395 (11)
C14—H14	0.9300	C43—H43	0.9300
C15—C16	1.378 (10)	C44—H44	0.9300
C15—C20	1.387 (10)	C45—H45A	0.9600
C16—C17	1.397 (11)	C45—H45B	0.9600
C16—H16	0.9300	C45—H45C	0.9600
C17—C18	1.370 (12)	C46—H46A	0.9600
C17—H17	0.9300	C46—H46B	0.9600
C18—C19	1.366 (12)	C46—H46C	0.9600
C18—H18	0.9300		
			
P2—Cu1—P1	122.03 (8)	C15—C20—C19	121.5 (8)
P2—Cu1—S1	102.05 (8)	C15—C20—H20	119.2
P1—Cu1—S1	112.28 (8)	C19—C20—H20	119.2
P2—Cu1—Cl1	108.42 (7)	C22—C21—C26	117.5 (8)
P1—Cu1—Cl1	103.10 (7)	C22—C21—P1	122.7 (7)
S1—Cu1—Cl1	108.54 (7)	C26—C21—P1	119.8 (6)
C9—P1—C15	104.5 (4)	C21—C22—C23	119.9 (9)
C9—P1—C21	100.1 (4)	C21—C22—H22	120.1
C15—P1—C21	102.9 (3)	C23—C22—H22	120.1
C9—P1—Cu1	115.9 (3)	C24—C23—C22	120.4 (9)
C15—P1—Cu1	113.8 (3)	C24—C23—H23	119.8
C21—P1—Cu1	117.7 (3)	C22—C23—H23	119.8
C39—P2—C33	102.5 (4)	C25—C24—C23	119.9 (9)
C39—P2—C27	102.6 (4)	C25—C24—H24	120.1
C33—P2—C27	103.5 (4)	C23—C24—H24	120.1
C39—P2—Cu1	114.8 (3)	C24—C25—C26	120.0 (9)
C33—P2—Cu1	114.7 (3)	C24—C25—H25	120.0
C27—P2—Cu1	117.0 (3)	C26—C25—H25	120.0
C1—S1—Cu1	109.2 (3)	C25—C26—C21	122.3 (8)
C1—N1—C3	110.3 (6)	C25—C26—H26	118.9
C1—N1—H1	124.8	C21—C26—H26	118.9
C3—N1—H1	124.8	C28—C27—C32	119.0 (8)
C1—N2—C2	111.3 (6)	C28—C27—P2	117.6 (7)
C1—N2—H2	124.3	C32—C27—P2	123.4 (7)
C2—N2—H2	124.3	C27—C28—C29	120.9 (8)
C5—O1—C8	117.7 (7)	C27—C28—H28	119.6
C45—O2—H2A	109.5	C29—C28—H28	119.6
C46—O3—H3	109.5	C30—C29—C28	118.3 (9)
N2—C1—N1	105.9 (7)	C30—C29—H29	120.8
N2—C1—S1	128.1 (6)	C28—C29—H29	120.8
N1—C1—S1	126.0 (6)	C29—C30—C31	122.0 (9)
N2—C2—C7	134.3 (8)	C29—C30—H30	119.0
N2—C2—C3	106.1 (7)	C31—C30—H30	119.0
C7—C2—C3	119.5 (8)	C30—C31—C32	119.4 (9)
C4—C3—C2	123.0 (7)	C30—C31—H31	120.3
C4—C3—N1	130.7 (7)	C32—C31—H31	120.3
C2—C3—N1	106.3 (7)	C27—C32—C31	120.4 (9)
C3—C4—C5	117.0 (8)	C27—C32—H32	119.8
C3—C4—H4	121.5	C31—C32—H32	119.8
C5—C4—H4	121.5	C38—C33—C34	118.4 (8)
O1—C5—C4	124.1 (8)	C38—C33—P2	118.9 (6)
O1—C5—C6	115.9 (7)	C34—C33—P2	122.7 (7)
C4—C5—C6	120.0 (8)	C35—C34—C33	119.2 (9)
C7—C6—C5	122.8 (8)	C35—C34—H34	120.4
C7—C6—H6	118.6	C33—C34—H34	120.4
C5—C6—H6	118.6	C36—C35—C34	121.1 (9)
C6—C7—C2	117.7 (8)	C36—C35—H35	119.4
C6—C7—H7	121.2	C34—C35—H35	119.4
C2—C7—H7	121.2	C37—C36—C35	119.8 (9)
O1—C8—H8A	109.5	C37—C36—H36	120.1
O1—C8—H8B	109.5	C35—C36—H36	120.1
H8A—C8—H8B	109.5	C36—C37—C38	119.8 (9)
O1—C8—H8C	109.5	C36—C37—H37	120.1
H8A—C8—H8C	109.5	C38—C37—H37	120.1
H8B—C8—H8C	109.5	C37—C38—C33	121.6 (8)
C14—C9—C10	116.9 (8)	C37—C38—H38	119.2
C14—C9—P1	119.0 (7)	C33—C38—H38	119.2
C10—C9—P1	124.1 (7)	C44—C39—C40	117.1 (8)
C11—C10—C9	119.0 (9)	C44—C39—P2	125.1 (7)
C11—C10—H10	120.5	C40—C39—P2	117.8 (6)
C9—C10—H10	120.5	C39—C40—C41	121.4 (8)
C12—C11—C10	120.4 (10)	C39—C40—H40	119.3
C12—C11—H11	119.8	C41—C40—H40	119.3
C10—C11—H11	119.8	C42—C41—C40	120.1 (9)
C11—C12—C13	122.8 (11)	C42—C41—H41	120.0
C11—C12—H12	118.6	C40—C41—H41	120.0
C13—C12—H12	118.6	C41—C42—C43	120.3 (10)
C12—C13—C14	117.6 (10)	C41—C42—H42	119.8
C12—C13—H13	121.2	C43—C42—H42	119.8
C14—C13—H13	121.2	C42—C43—C44	120.7 (10)
C9—C14—C13	123.2 (9)	C42—C43—H43	119.7
C9—C14—H14	118.4	C44—C43—H43	119.7
C13—C14—H14	118.4	C39—C44—C43	120.4 (9)
C16—C15—C20	117.6 (8)	C39—C44—H44	119.8
C16—C15—P1	124.4 (7)	C43—C44—H44	119.8
C20—C15—P1	118.0 (7)	O2—C45—H45A	109.5
C15—C16—C17	120.6 (8)	O2—C45—H45B	109.5
C15—C16—H16	119.7	H45A—C45—H45B	109.5
C17—C16—H16	119.7	O2—C45—H45C	109.5
C18—C17—C16	120.9 (9)	H45A—C45—H45C	109.5
C18—C17—H17	119.5	H45B—C45—H45C	109.5
C16—C17—H17	119.5	O3—C46—H46A	109.5
C19—C18—C17	119.1 (9)	O3—C46—H46B	109.5
C19—C18—H18	120.4	H46A—C46—H46B	109.5
C17—C18—H18	120.4	O3—C46—H46C	109.5
C18—C19—C20	120.3 (9)	H46A—C46—H46C	109.5
C18—C19—H19	119.9	H46B—C46—H46C	109.5
C20—C19—H19	119.9		
			
P2—Cu1—P1—C9	177.7 (3)	Cu1—P1—C15—C20	48.6 (7)
S1—Cu1—P1—C9	−60.8 (3)	C20—C15—C16—C17	0.1 (12)
Cl1—Cu1—P1—C9	55.9 (3)	P1—C15—C16—C17	−180.0 (6)
P2—Cu1—P1—C15	−61.1 (3)	C15—C16—C17—C18	0.5 (13)
S1—Cu1—P1—C15	60.4 (3)	C16—C17—C18—C19	−0.6 (14)
Cl1—Cu1—P1—C15	177.0 (3)	C17—C18—C19—C20	0.2 (14)
P2—Cu1—P1—C21	59.3 (3)	C16—C15—C20—C19	−0.5 (12)
S1—Cu1—P1—C21	−179.2 (3)	P1—C15—C20—C19	179.5 (6)
Cl1—Cu1—P1—C21	−62.6 (3)	C18—C19—C20—C15	0.4 (13)
P1—Cu1—P2—C39	177.6 (3)	C9—P1—C21—C22	102.6 (8)
S1—Cu1—P2—C39	51.4 (3)	C15—P1—C21—C22	−4.9 (8)
Cl1—Cu1—P2—C39	−63.0 (3)	Cu1—P1—C21—C22	−130.8 (7)
P1—Cu1—P2—C33	59.4 (3)	C9—P1—C21—C26	−76.0 (7)
S1—Cu1—P2—C33	−66.9 (3)	C15—P1—C21—C26	176.5 (7)
Cl1—Cu1—P2—C33	178.7 (3)	Cu1—P1—C21—C26	50.5 (7)
P1—Cu1—P2—C27	−62.1 (3)	C26—C21—C22—C23	2.5 (13)
S1—Cu1—P2—C27	171.7 (3)	P1—C21—C22—C23	−176.2 (7)
Cl1—Cu1—P2—C27	57.2 (3)	C21—C22—C23—C24	−0.7 (15)
P2—Cu1—S1—C1	−138.7 (3)	C22—C23—C24—C25	−2.1 (16)
P1—Cu1—S1—C1	89.0 (3)	C23—C24—C25—C26	2.8 (15)
Cl1—Cu1—S1—C1	−24.3 (3)	C24—C25—C26—C21	−0.9 (14)
C2—N2—C1—N1	−1.4 (8)	C22—C21—C26—C25	−1.8 (13)
C2—N2—C1—S1	177.4 (6)	P1—C21—C26—C25	176.9 (7)
C3—N1—C1—N2	1.1 (8)	C39—P2—C27—C28	153.8 (6)
C3—N1—C1—S1	−177.7 (6)	C33—P2—C27—C28	−99.9 (7)
Cu1—S1—C1—N2	−168.8 (6)	Cu1—P2—C27—C28	27.3 (7)
Cu1—S1—C1—N1	9.8 (7)	C39—P2—C27—C32	−28.0 (7)
C1—N2—C2—C7	179.0 (8)	C33—P2—C27—C32	78.3 (7)
C1—N2—C2—C3	1.1 (8)	Cu1—P2—C27—C32	−154.5 (6)
N2—C2—C3—C4	−178.2 (7)	C32—C27—C28—C29	−1.8 (12)
C7—C2—C3—C4	3.5 (12)	P2—C27—C28—C29	176.5 (6)
N2—C2—C3—N1	−0.4 (8)	C27—C28—C29—C30	1.2 (13)
C7—C2—C3—N1	−178.7 (7)	C28—C29—C30—C31	−0.1 (13)
C1—N1—C3—C4	177.2 (8)	C29—C30—C31—C32	−0.3 (14)
C1—N1—C3—C2	−0.4 (8)	C28—C27—C32—C31	1.4 (12)
C2—C3—C4—C5	−2.0 (12)	P2—C27—C32—C31	−176.8 (6)
N1—C3—C4—C5	−179.2 (7)	C30—C31—C32—C27	−0.4 (13)
C8—O1—C5—C4	−10.8 (12)	C39—P2—C33—C38	−75.4 (7)
C8—O1—C5—C6	169.2 (8)	C27—P2—C33—C38	178.2 (7)
C3—C4—C5—O1	179.4 (7)	Cu1—P2—C33—C38	49.6 (7)
C3—C4—C5—C6	−0.5 (12)	C39—P2—C33—C34	107.1 (7)
O1—C5—C6—C7	−178.4 (8)	C27—P2—C33—C34	0.7 (8)
C4—C5—C6—C7	1.5 (13)	Cu1—P2—C33—C34	−127.9 (6)
C5—C6—C7—C2	−0.1 (13)	C38—C33—C34—C35	−0.3 (13)
N2—C2—C7—C6	180.0 (8)	P2—C33—C34—C35	177.2 (7)
C3—C2—C7—C6	−2.3 (11)	C33—C34—C35—C36	0.9 (15)
C15—P1—C9—C14	−104.9 (6)	C34—C35—C36—C37	−2.2 (16)
C21—P1—C9—C14	148.8 (6)	C35—C36—C37—C38	2.7 (15)
Cu1—P1—C9—C14	21.1 (7)	C36—C37—C38—C33	−2.1 (14)
C15—P1—C9—C10	75.4 (7)	C34—C33—C38—C37	0.9 (13)
C21—P1—C9—C10	−30.9 (7)	P2—C33—C38—C37	−176.7 (7)
Cu1—P1—C9—C10	−158.6 (6)	C33—P2—C39—C44	−15.7 (9)
C14—C9—C10—C11	−1.0 (11)	C27—P2—C39—C44	91.4 (8)
P1—C9—C10—C11	178.7 (6)	Cu1—P2—C39—C44	−140.6 (7)
C9—C10—C11—C12	0.9 (14)	C33—P2—C39—C40	165.6 (6)
C10—C11—C12—C13	−0.4 (16)	C27—P2—C39—C40	−87.3 (7)
C11—C12—C13—C14	0.0 (16)	Cu1—P2—C39—C40	40.7 (7)
C10—C9—C14—C13	0.6 (12)	C44—C39—C40—C41	−2.0 (13)
P1—C9—C14—C13	−179.1 (7)	P2—C39—C40—C41	176.8 (7)
C12—C13—C14—C9	−0.1 (14)	C39—C40—C41—C42	2.3 (14)
C9—P1—C15—C16	−4.0 (8)	C40—C41—C42—C43	−0.4 (17)
C21—P1—C15—C16	100.2 (7)	C41—C42—C43—C44	−1.8 (18)
Cu1—P1—C15—C16	−131.3 (6)	C40—C39—C44—C43	−0.3 (14)
C9—P1—C15—C20	176.0 (6)	P2—C39—C44—C43	−178.9 (8)
C21—P1—C15—C20	−79.8 (6)	C42—C43—C44—C39	2.2 (17)

### Hydrogen-bond geometry (Å, º)

**Table:**

*D*—H···*A*	*D*—H	H···*A*	*D*···*A*	*D*—H···*A*
N1—H1···Cl1	0.86	2.30	3.136 (6)	165
N2—H2···O2	0.86	2.08	2.893 (9)	157
O2—H2*A*···O3^i^	0.82	2.00	2.728 (10)	148
O3—H3···Cl1^ii^	0.82	2.35	3.170 (8)	176

Symmetry codes: (i) −*x*+1, −*y*+1, −*z*; (ii) *x*+1, *y*, *z*.
